# A Comparative Study of Nanobio Interaction of Zn-Doped CdTe Quantum Dots with Lactoferrin Using Different Spectroscopic Methods

**DOI:** 10.3390/ijms24119325

**Published:** 2023-05-26

**Authors:** Meng Ji, Liwei Ren, Chunyuan Tian, Xuming Zhuang, Feng Luan

**Affiliations:** College of Chemistry and Chemical Engineering, Yantai University, Yantai 264005, China; jm13156835210@163.com (M.J.); q602842472@gmail.com (L.R.); cytian@ytu.edu.cn (C.T.); xmzhuang@iccas.ac.cn (X.Z.)

**Keywords:** quantum dots (QDs), lactoferrin (LF), interaction, spectroscopic methods, thermodynamic constants, nano-effects

## Abstract

In this paper, glutathione (GSH)-coated Zn-doped CdTe quantum dots (QDs) with different particle sizes were synthesized using the “reflow method”, and the interaction mechanism between the two QDs and lactoferrin (LF) was investigated systemically with different spectroscopic methods. The steady-state fluorescence spectra showed that the LF formed a tight complex with the two QDs through static bursting and that the electrostatic force was the main driving force between the two LF–QDs systems. The complex generation process was found to be spontaneous (ΔG < 0) and accompanied by exothermic and increasing degrees of freedom (ΔH < 0, ΔS > 0) by using the temperature-dependent fluorescence spectroscopy. The critical transfer distance (R_0_) and donor–acceptor distance (r) of the two LF–QDs systems were obtained based on the fluorescence resonance energy transfer theory. In addition, it was observed that the QDs changed the secondary and tertiary structures of LF, leading to an increase in the hydrophobicity of LF. Further, the nano-effect of orange QDs on LF is much larger than that of green QDs. The above results provide a basis for metal-doped QDs with LF in safe nano-bio applications.

## 1. Introduction

Quantum dots (QDs), as an important low-dimensional semiconductor material, have been applied in a wide range of fields, including biosensors, environmental monitoring, photovoltaic cells, tumor targeting, and biomedical imaging [1,2,3]. In these applications, Cd-based nanomaterials play an important role due to their excellent properties such as high fluorescence yield, broad excitation spectrum, narrow emission spectrum, and high photostability. However, this nanomaterial poses a potential hazard to the environment and human health in terms of safety applications due to its own heavy metal ion release and surface ligand effects [4,5]. 

Thus, it is important to balance the relationship between toxicity and excellent performance of this kind of material. Reducing the toxicity of QDs and improving their biocompatibility can be achieved through the surface modification of Cd-based QDs. In recent years, due to the inherent crystal surface defects of Cd-based QDs, the doping of transition metals can change the surface defect energy level, resulting in better optical properties and low cytotoxicity of QDs [6,7]. Safari et al. successfully synthesized water-soluble Ni-doped CdTe QDs using a facile, novel, and green method, and then established a fluorescence burst method using these QDs for the rapid determination of pyrazinamide (PZA) in plasma samples [8]. Moreover, Buchtelova et al. found that Ln-doped CdTe QDs not only have high colloidal stability as well as better optical properties but also significantly enhance their cytocompatibility [9]. Such Cd-based QDs doped with transition metal ions effectively reduced their cytotoxicity, thus improving their reliability for safe applications.

In order to be effectively used in the biomedical field, it is necessary to investigate the interaction between QDs and proteins. When QDs are placed in a physiological environment, proteins interact with QDs to produce a “protein corona” phenomenon, which changes the original surface properties of QDs, thus affecting the functional properties of QDs [10,11]. In addition, the interaction between QDs and proteins disrupts the original structure and functional realization of proteins, which in turn affects the normal function of the organism [12,13]. Therefore, an intensive study of the interactions between QDs and proteins is instructive for their biological effects.

In recent years, research on the interaction between QDs and proteins has become a popular topic. Kaur et al. performed spectrophotometry to demonstrate that the main binding mode of trypsin with water-soluble CdSe QDs is electrostatic interaction, and the combination of the two enhanced the luminescence intensity of trypsin in a certain concentration range, which is useful for determining the enzyme concentration of unknown samples [14]. Zhu et al. combined ZnSe QDs with three different surface modifications of L-glutathione (GSH), L-cysteine (Cys), and thioglycolic acid (TGA) with bovine serum proteins (BSA) and demonstrated the difference of unique surface modifications on their binding modes using spectroscopy and molecular simulation methods [15]. Wang’s team explored the interaction mechanism between CdTe QDs and transferrin (TF)—as well as the effect of QDs-TF complex formation on TF structure and the cytotoxic effect on primary kidney cells in mice—and elucidated the formation mechanism of QDs-TF complexes [16].

Lactoferrin (LF) is a non-heme, iron-binding protein that belongs to the transferrin family and is expressed and secreted by glandular cells. The protein is an 80 kDa glycosylated protein containing 703 amino acid residues with a high degree of homology among species, and its primary structure has been well characterized [17]. Since its discovery, LF and its related peptides have played an active role in a wide range of biological functions, not only as important non-specific host defense molecules against a variety of pathogens but also for immunomodulatory, anti-inflammatory, and antiviral properties, and the application of LF has attracted increasing attention [18,19]. So far, the interactions between proteins and QDs have been mainly directed to human serum albumin (HSA), BSA, trypsin, plasma proteins, etc., but few studies have been reported on the interactions between QDs and LF. As one of the most promising strategic proteins, LF combined with nanomaterials to form functional complexes can have enhanced functions, which can play a positive role in the utilization of LF. Therefore, exploring the interactions between doped QDs and LF will help to provide a deeper understanding of the potential toxicity risk to organisms at the molecular level and provide valid information.

In the present work, two glutathione (GSH)--coated Zn-doped CdTe QDs with different particle sizes were synthesized using the “reflow method”, and their interactions with LF were explored using different spectroscopic methods. The thermodynamic properties of the two LF–QDs systems were investigated with steady-state fluorescence spectroscopy, and the conformational changes of the LF–QDs systems were also observed using UV-Vis absorption spectroscopy, three-dimensional (3D) fluorescence spectroscopy, synchronous fluorescence spectroscopy, and circular dichroism (CD) spectroscopy. In this study, we tried to reveal the effects of different particle sizes of CdTe:Zn^2+^ QDs on LF and its conformational and functional changes and attempted to elucidate the biological properties of CdTe:Zn^2+^ QDs and their biological effects. Meanwhile, it provides a theoretical basis for the integrated application of metal-doped QDs with LF.

## 2. Results and Discussion

### 2.1. Characterization of CdTe: Zn^2+^ QDs

**Figure 1:**
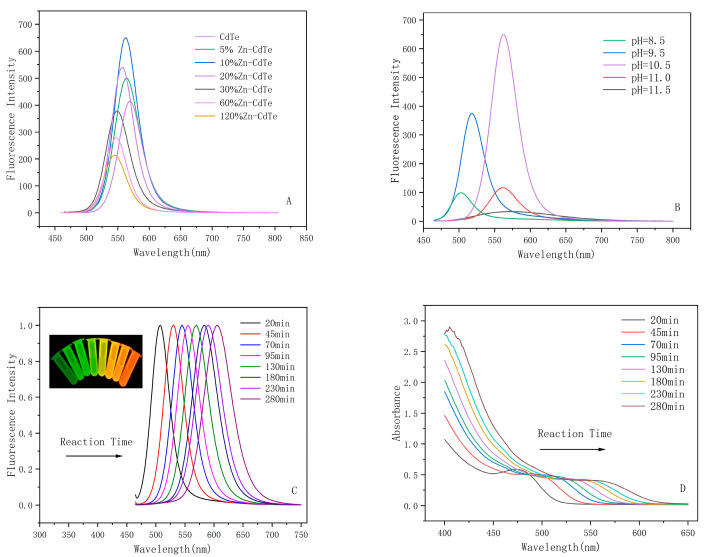
(**A**) Fluorescence intensity at different Zn^2+^ doping ratios (Reaction time = 95min). (**B**) Fluorescence spectra at different pH conditions under 10% Zn^2+^ conditions (Reaction time = 95 min). (**C**) Normalized fluorescence spectra of QDs at different reaction times; the inset shows the image of QDs under UV lamp. (**D**) UV-Vis absorption spectra of QDs at different reaction times.

Figure 1A shows the fluorescence intensities of different Zn^2+^ doping ratios. As can be seen from the figure, the fluorescence intensity reaches the maximum value when Zn/Cd = 1/10, and its fluorescence quantum yield (FLQY) increases about 17.15% compared to that of the undoped CdTe QDs. (The FLQY was 45.99% for CdTe QDs and 63.14% for CdTe:Zn^2+^ QDs.) Due to the low doping of Zn^2+^, the fluorescence defects on the surface of CdTe QDs are filled, which leads to the improvement of their optical properties. The pH has a large influence on the synthesis of QDs; Figure 1B shows the fluorescence intensity under 10% Zn^2+^ doped CdTe QDs at different pH conditions. The most advantageous condition for the synthesis of QDs was pH = 10.5. Therefore, we chose 10% Zn^2+^ doping and pH = 10.5 for the following study. The fluorescence spectra (Figure 1C) and UV-Vis absorption spectra (Figure 1D) of CdTe:Zn^2+^ QDs show that the absorption peaks as well as the wavelength of the fluorescence emission peaks of the QDs undergo a significant red shift with increasing reaction time, which indicates that the size of the QDs increases with increasing reflow time.

**Figure 2:**
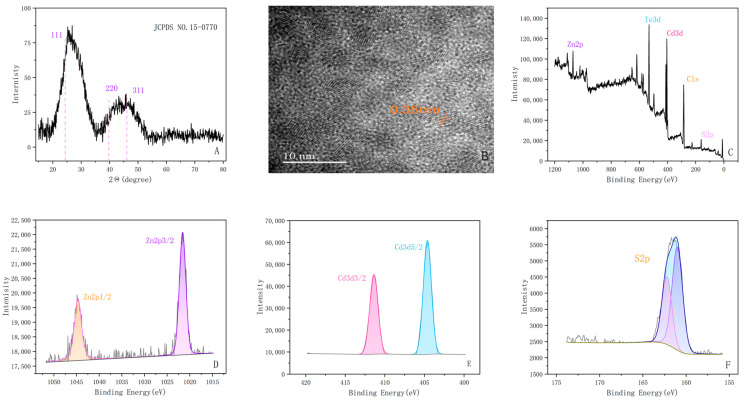
Characteristics of CdTe:Zn^2+^ QDs (Reaction Time = 95min). (**A**) XRD plots. (**B**) HRTEM image at 10 nm scale. (**C**–**F**) Zn2p, Te3d, Cd3d, C1s, and the XPS plot of S2p.

As shown in the XRD plot of QDs in Figure 2A, the synthesized QDs correspond to the three crystallographic planes data of the standard card of CdTe QDs (JCPDS NO. 15-0770), which indicates that the doping of Zn^2+^ does not affect the original bulk cubic CdTe structure. In addition, the HRTEM image of the CdTe:Zn^2+^ QDs (Figure 2B) shows that the lattice planes as well as the lattice distance (0.35 nm) correspond to the planes in XRD (111), which affirms that the synthesis of the QDs was successful. XPS is meaningful for the analysis of the QDs surface structure, and Figure 2C–F shows the presence of Zn2p, S2p, Cd3d, Te3d, and other peaks. The Zn2p peak appears at 1021.46 ev and 1044.79 ev, while for the S2p peak, the peaks at 161.46ev and 162.53ev correspond to the typical peaks of Cd-S and Cd-SR, respectively.

### 2.2. Fluorescence Spectroscopy Study of Interaction between CdTe:Zn^2+^ QDs and LF

#### 2.2.1. Fluorescence Quenching Mechanism

**Figure 3:**
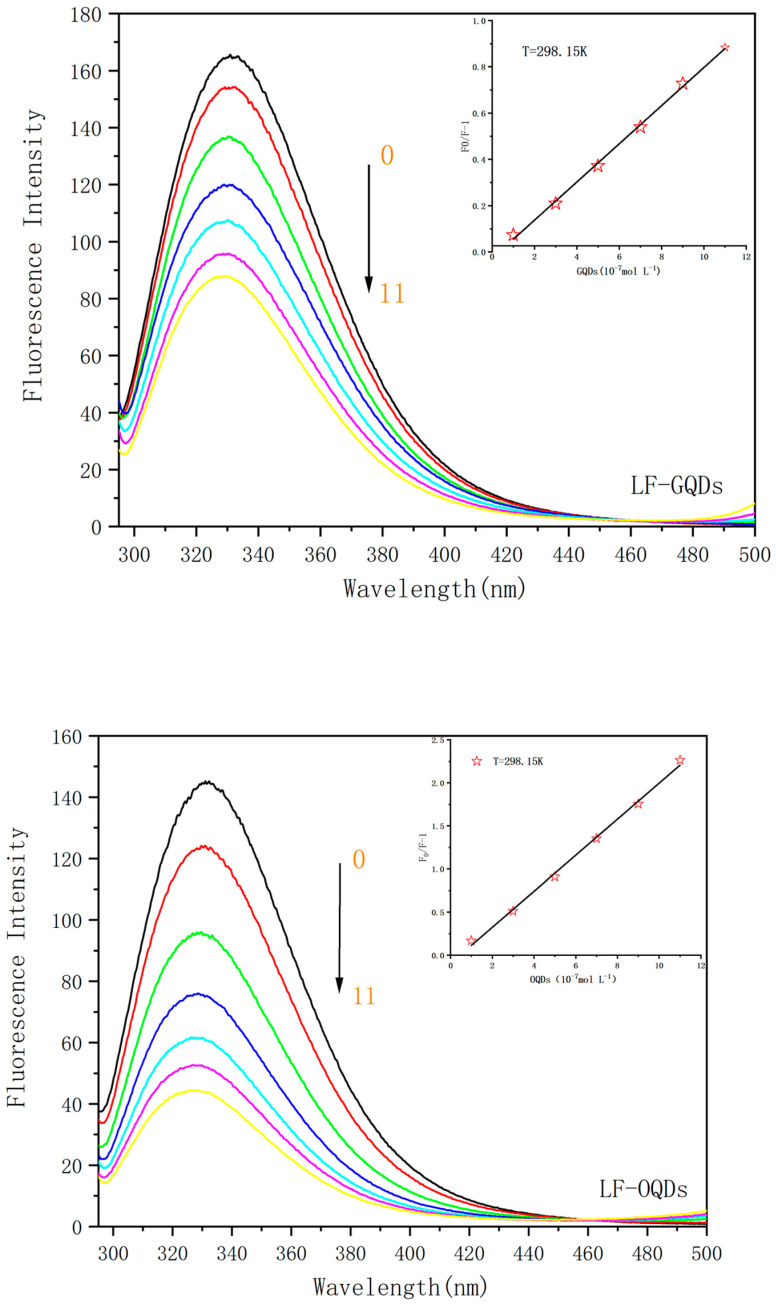
Effect of two types of CdTe: Zn^2+^ QDs on the fluorescence spectrum of LF. The insertion corresponds to 298.15 K. C(LF) = 1.0 × 10^−6^ mol L^−1^; C (GQDs)/(0,1,3,5,7,9,11 × 10^−7^ mol L^−1^), C (OQDs)/(0,1,3,5,7,9,11 × 10^−7^ mol L^−1^).

In order to investigate whether the interaction between QDs and LF occurs, QDs with reaction times of 70 min (Green-QDs) as well as 230 min (Orange-QDs) were chosen in the following studies. According to Peng’s method [20], the particle diameter (nm) of the QDs was estimated from the first excitation absorption peak of the UV-Vis absorption spectrum; the diameters of GQDs and OQDs are 2.45 nm and 3.15 nm, respectively. Two different concentrations of CdTe:Zn^2+^ QDs were added sequentially to 10^−6^ mol/L LF and incubated at three different temperatures (298.15 K, 305.15 K, 313.15 K), after which their fluorescence spectra were recorded and shown in Figure 3. From the figure, one can see that not only LF but also the two LF–QDs systems exhibited strong fluorescence emission at 330 nm under the excitation wavelength of 280 nm. The fluorescence intensity of both LF–QDs systems decreased sequentially with the increase of QDs concentration, indicating the existence of a strong interaction between the QDs and LF.

It has been proven that proteins have endogenous fluorescence within them. When the interaction between QDs and LF occurs, it is often accompanied by reactions such as energy transfer, molecular rearrangement, and the formation of steady-state complexes resulting in changes in the endogenous fluorescence of the protein [21,22]. The fluorescence burst mechanism can be divided into three cases: static burst, dynamic burst, and combined dynamic and static burst mechanism. In the dynamic burst process, the increase in temperature leads to an increase in the collisional diffusion coefficient, so the burst constant is negatively correlated with temperature; for the static burst process, the increase in temperature is detrimental to the stability of the steady-state complex, so the burst constant is positively correlated with temperature [23]. For the determination of the burst mechanism, it can be calculated with the Stern–Volmer equation [24]:

(1) $$\frac{F_{0}}{F}=1+K_{Sv}\left[Q\right]=1+K_{q}\tau_{0}\left[Q\right]$$

where *F* and *F*_0_ represent the fluorescence intensity of LF with and without the presence of QDs, respectively; the *K_SV_* represents the Stern–Volmer burst constant; the [*Q*] is the QDs concentration; the *K_q_* represents the bimolecular burst rate constant; and the *τ_0_* refers to the fluorescence lifetime of LF in the presence of no QDs.

**Figure 4:**
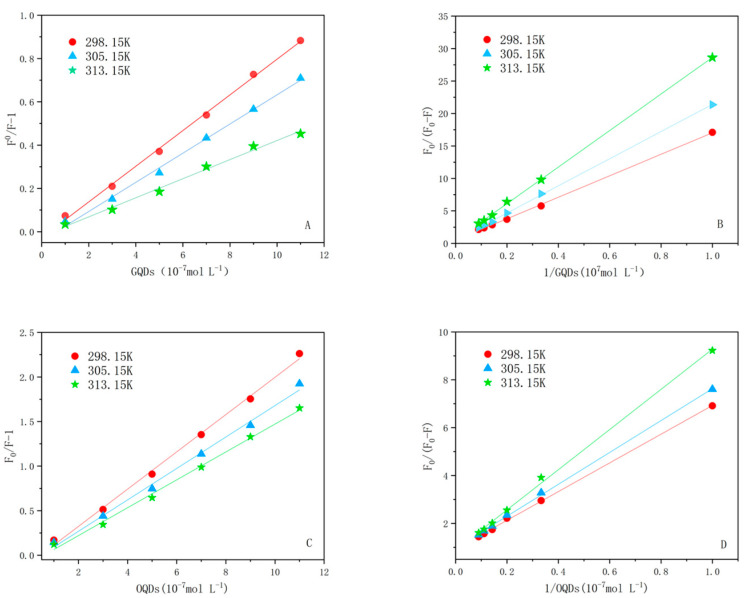
(**A**,**C**) are the Stern–Volmer plots and modified Stern–Volmer plots of LF–GQDs system, respectively. (**B**,**D**) are the Stern–Volmer plots and modified Stern–Volmer plots of LF–GQDs system, respectively.

The Stern–Volmer plots of the two QDs interacting with LF are shown in Figure 4. The corresponding fitted parameters are listed in Table 1. Both *K_sv_* and *K_q_* show a negative correlation with temperature, and their *K_q_* constants are much larger than the maximum *K_q_* value for dynamic burst (2.0 × 10 L mol^−1^s^−1^n) [25]. Thus, the burst mechanism of both LF–QDs systems is static burst. In addition, the comparison of *K_sv_* and *K_q_* of LF–OQDs systems with LF–GQDs systems confirmed that the former system has greater bursting ability than the later one.

Further, for the static burst process, other parameters of the system can be obtained with the modified Stern–Volmer equation [26]:

(2) $$\frac{F_{0}}{\Delta F}=\frac{1}{f_{a}K_{a}\left[Q\right]}+\frac{1}{f_{a}}$$

where Δ*F* represents the different fluorescence intensity of the fluorescent molecules before and after the addition of QDs, *K_a_* is the associative binding constant, *f_a_* is the solvent accessible for the molar fraction of fluorophores.

The linear relationship between *F*_0_*/*Δ*F* and [*Q*]^−1^ for the two LF–QDs systems at a certain CdTe:Zn^2+^ QD concentration is shown in Figure 4, and the *K_a_* values for the two LF–QDs systems are listed in Table 1. The *K_a_* and *K_sv_* values decrease with increasing temperature in the interaction of proteins with QDs, which indicates that the fluorescence burst mechanism of the two LF–QDs systems is a static burst mechanism, the same as mentioned above. In addition, the K_a_ values of LF–OQDs systems are larger than those of LF–GQDs systems at the same temperature, which indicates that OQDs are far more advantageous than GQDs in the binding of QDs to LF.

#### 2.2.2. Binding Constant and Binding Number

The binding constants (*K_b_*) and the number of binding sites (*n*) can be calculated from the Scatchard equation [27]:

(3) $$\log\frac{F_{0}-F}{F}=\log K_{b}+n\log\left[Q\right]$$

where *K_b_* is the binding constant and *n* is the number of binding sites. *F* and *F*_0_ have the same meaning as above.

**Figure 5:**
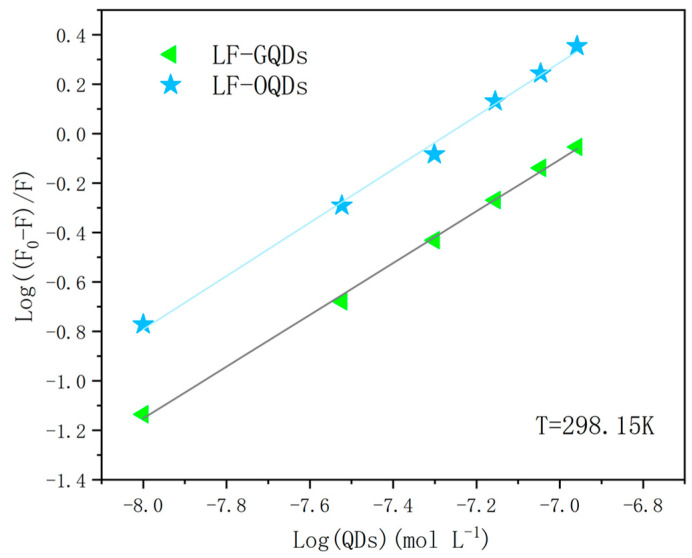
The Hill plots of two LF–QDs systems at 298.15 K.

Figure 5 shows the double logarithmic curves of the two QDs bursting LF fluorescence at 298.15 K for different QD concentrations. As shown in Table 2, the binding sites of the two LF–QDs systems are about 1, which indicates that the two QDs bind strongly with LF in a 1:1 molar ratio. From the binding constants of the two LF–QDs systems, it is known that both QDs can strongly interact with LF, but OQDs possess a greater binding probability than GQDs.

#### 2.2.3. Binding Force

Proteins interact with QDs by means of hydrogen bonds, van der Waals forces, electrostatic forces, etc. [28,29]. In order to obtain information related to the interaction of LF with two types of QDs, we calculated the corresponding thermodynamic parameters using the Van’t Hoff equation [30]:

(4) $$\ln K_{a}=-\frac{\Delta H}{RT}+\frac{\Delta S}{R}$$

where *K_a_* is the associative binding constant for the interaction process at the corresponding temperature and *R* is the universal gas constant.

**Figure 6:**
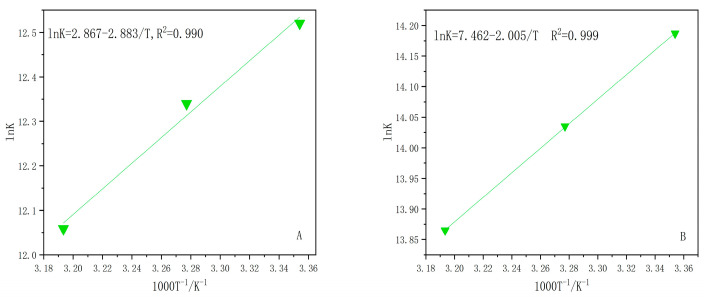
The Van’t Hoff plots of two LF–QDs systems,(**A**): LF-GQDs systems; (**B**): LF-OQDs systems.

As shown in Figure 6, plotted with *lnK_a_* against 1000 T^−1^/K^−1^, the two LF–QDs systems show a good linear relationship. The Gibbs free energy (Δ*G*) of the interaction process can be obtained using the following equation [30]:

(5) ΔG=ΔH−TΔS

As shown in the thermodynamic parameters of the two LF–QDs systems in Table 3, the interaction processes of both LF–QDs systems are spontaneous (Δ*G* < 0) and are accompanied by an exothermic reaction and increasing degrees of freedom (Δ*H* < 0, Δ*S* > 0). Therefore, the process of interaction, mainly under the action of electrostatic force, transforms QDs from a solvent-free state to a state tightly bound to LF. In addition, during the interaction of OQDs and GQDs with LF, the altered nano-effects make the electrostatic force of the former much larger than that of the latter.

Under normal physiological pH conditions, the zeta potential values of LF and the two QDs were tested, and the results are +2.5 mv, -6.9 mv, and −10.0 mv, respectively. LF, being a basic protein (with an iso-electric point of 8.5-9.2), should have a positive surface charge under these conditions, which is consistent with the above results. Therefore, there is an electrostatic force in the process of their interaction.

**Figure 7:**
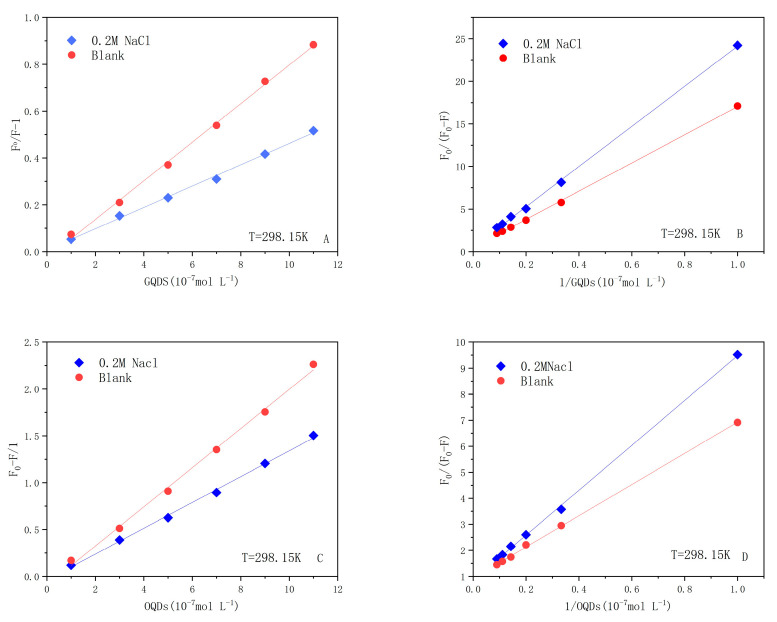
The two LF–QDs–NaCl systems (**A**,**C**) Stern–Volmer plots and modified Stern–Volmer plots (**B**,**D**) at 298.15 K.

The effect of strong electrolyte environment on the electrostatic forces is particularly prominent; thus, in this work, the two LF–QDs systems were placed in 0.2 M NaCl solution. It is observed from Figure 7 and Table 4 that both *K_sv_* and *K_a_* of the LF–QDs system decreased to different degrees in 0.2M NaCl solution, while the decrease was more prominent in the OQDs–LF system. This also demonstrates the effect of nanoscale effect on the binding force.

#### 2.2.4. Binding Distance

According to the fluorescence resonance energy transfer (FRET) theory, when the fluorescence emitted by the donor can be absorbed by the acceptor and the interaction distance between the two is less than 7 nm, it will cause the energy transfer phenomenon to occur [31]. The burst phenomenon after the binding of LF and QDs indicates that an energy transfer phenomenon is generated. Therefore, for the binding distance (*r*) and energy transfer efficiency (*E*) between both LF–QDs systems can be calculated using the following equation [32]:

(6) $$E=1-\frac{F}{F_{0}}=\frac{R_{0}^{6}}{R_{0}^{6}+r^{6}}$$

where *E* is the energy efficiency, *r* is the interaction distance between QDs and LF, and *R*_0_ is the critical distance when the energy transfer efficiency reaches 50% during the interaction. For *R*_0_, the calculation can be performed with the following equation [32]:

(7) $$R_{0}^{6}=8.79\times 10^{-25}K^{2}n^{-4}\Phi J$$

The *K*^2^ indicates the orientation factor of the random distribution between the QDs and LF, n is the refractive index (also called refractive index) of the medium in which it is located, Φ represents the fluorescence quantum yield of LF, and *J* is the overlap integral between the emission spectrum of LF and the UV absorption spectrum of the QDs. For the acquisition of *J*, we can do the following equation [32]:

(8) $$J=\frac{\int_{0}^{\infty }F\left(\lambda \right)\varepsilon \left(\lambda \right)\lambda^{4}d\lambda }{\int_{0}^{\infty }F\left(\lambda \right)d\lambda }$$

The *F*(*λ*) denotes the fluorescence intensity value of LF at *λ* wavelength, and *ε*(*λ*) is the molar absorption coefficient of QDs at *λ* wavelength.

**Figure 8:**
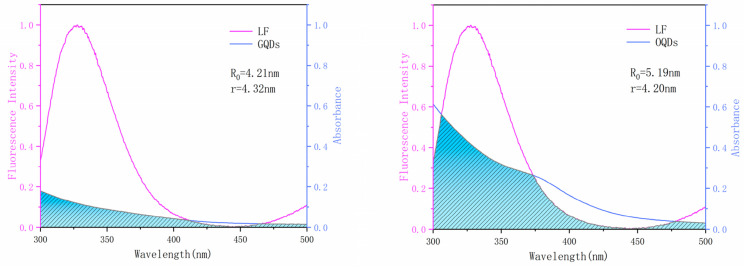
The UV-Vis absorption spectra of the two LF–QDs systems overlapped with the fluorescence spectra of LF.

The overlapping integral plots of the two LF–QDs systems are shown in Figure 8. The average binding distances of both LF–QDs systems are below 7 nm, which is consistent with the non-radiative energy transfer in the interaction process. In addition, OQDs are closer to the tryptophan residues of LF than GQDs, which makes OQDs possess a more powerful bursting ability.

### 2.3. UV-Vis Absorption Spectroscopy Study of Interaction between CdTe:Zn^2+^ QDs and LF

**Figure 9:**
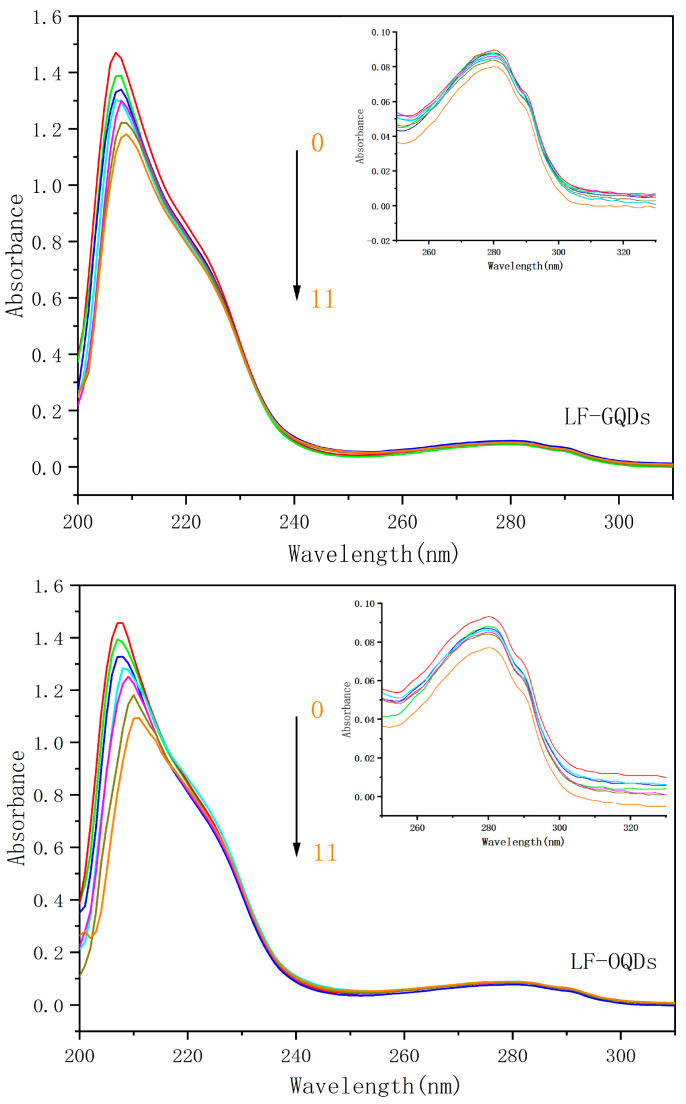
The UV-Vis absorption spectra of two LF–QDs. C_(LF)_ = 1.0 × 10^−6^ mol L^−1^; C_(GQDs)_/(0,1,3,5,7,9,11 × 10^−7^ mol L^−1^), C_(OQDs)_/(0,1,3,5,7,9,11 × 10^−7^ mol L^−1^).

UV-Vis absorption spectroscopy is a common method to study the structural changes of proteins during the interaction [33]. The UV-Vis absorption spectra of the two LF–QDs systems are shown in Figure 9. With the increase of the QDs concentration, the intensity of the absorption peak of LF shows a decreasing trend and a red shift at the strong absorption peak at about 208 nm, which indicates that the peptide structure of LF is changed. Meanwhile, the absorption peak at 278 nm possesses a smaller change, which indicates that the micro-environment of the chromophore of LF is slightly changed [34]. Therefore, it can be concluded that the interaction of LF–QDs leads to the formation of steady-state complexes, which again proves that the burst mechanism between LF–QDs is a static burst. 

In the comparison of the interaction between OQDs, GQDs, and LF, the effect of OQDs on LF is much greater than that of GQDs in both the alteration of peptide structure and the destruction of LF tertiary structure.

### 2.4. Synchronous Fluorescence Spectroscopy Study of Interaction between CdTe:Zn^2+^ QDs and LF

**Figure 10:**
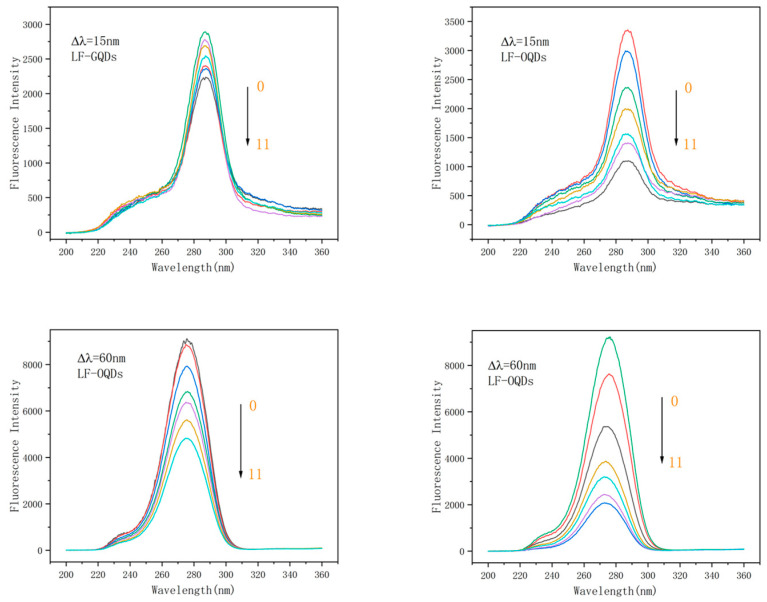
Synchronous fluorescence spectra of two different LF–QDs systems. C_(LF)_ = 1.0 × 10^−6^ mol L^−1^; C_(GQDs)_/(0,1,3,5,7,9,11 × 10^−7^ mol L^−1^); C_(OQDs)_/(0,1,3,5,7,9,11 × 10^−7^ mol L^−1^).

The change of the protein micro-environment during the LF–QDs interaction can be studied with synchrotron fluorescence spectroscopy. When Δλ is fixed at 15 nm and 60 nm, it reveals information about the micro-environment of tyrosine residues and trypsin residues. [35]. The synchronous fluorescence spectra of the two LF–QDs systems were shown in Figure 10. The fluorescence intensities of both tyrosine and tryptophan residues were burst by CdTe:Zn^2+^ QDs, and the extent of the burst increased gradually with the increase of QDs concentration. At the same time, tryptophan residues were subjected to much greater bursts of QDs than tyrosine residues compared to both, this suggests that QDs are closer to the vicinity of tryptophan residues in the binding process of LF. In addition, the positions of the characteristic peaks of tyrosine for both LF–QDs systems did not change greatly with the increase of QDs concentration, indicating that the micro-environment of tyrosine residues did not change drastically in the presence of both QDs. While for tryptophan residues, the characteristic peaks were slightly blue-shifted, indicating that the presence of QDs decreased the polarity of the micro-environment around tryptophan residues and increased the hydrophobicity; thus, it had altered the tertiary structure of LF.

### 2.5. Three-Dimensional Fluorescence Spectrometry Study of Interaction between CdTe:Zn^2+^ QDs and LF

**Figure 11:**
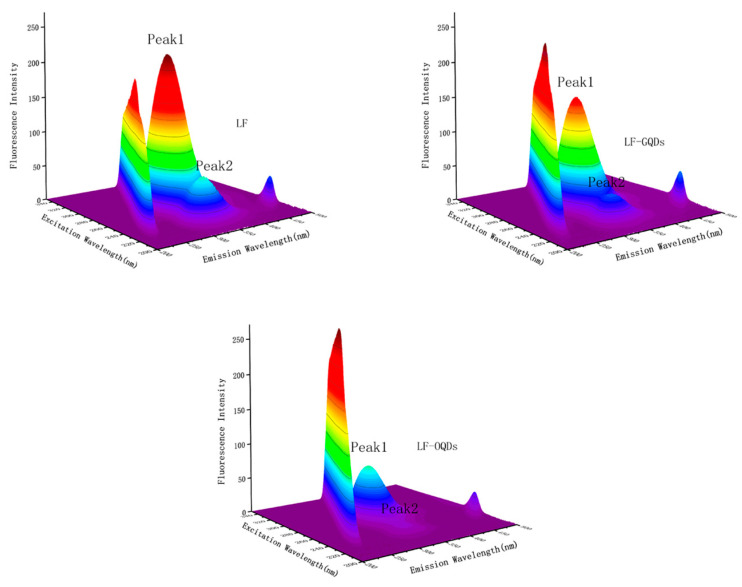
Three-dimensional fluorescence spectra of LF and two LF–QDs systems. C_(LF)_ = 2 × 10^−6^ mol L^−1^; C_(GQDs)_ = C_(OQDs)_ =5.0 × 10^−7^ mol L^−1^.

It has been proven that 3D fluorescence spectrometry can give the information of the conformational changes of LF according to fluorescence characteristics such as the shift of the excitation wavelength or the emission wavelength of fluorescence peaks or the appearance of new fluorescence peaks [36]. The results of this systems are shown in Figure 11 and Table 5. In the figures, Peak1 represents the endogenous fluorescence characteristics of tyrosine and tryptophan residues in LF, which mainly reflect the changes of protein tertiary structure; Peak2 shows the fluorescence characteristics of LF peptide backbone structure, which mainly reflects the changes of protein secondary structure. From Figure 11 and Table 5, it can be seen that by adding the CdTe:Zn^2+^ QDs, not only did the fluorescence intensity of the two fluorescence features of LF decreased but also the fluorescence position changed. Further, the experimental results show that the interaction between the two CdTe:Zn^2+^ QDs and LF has different effects on the secondary and tertiary structures of LF. That is, the OQDs are far more influential than the GQDs for the degree of unfolding of LF polypeptides and the enhancement of hydrophobicity in the micro-environment around the tryptophan residues.

### 2.6. Circular Dichroism (CD) Study of Interaction between CdTe:Zn^2+^ QDs and LF

**Figure 12:**
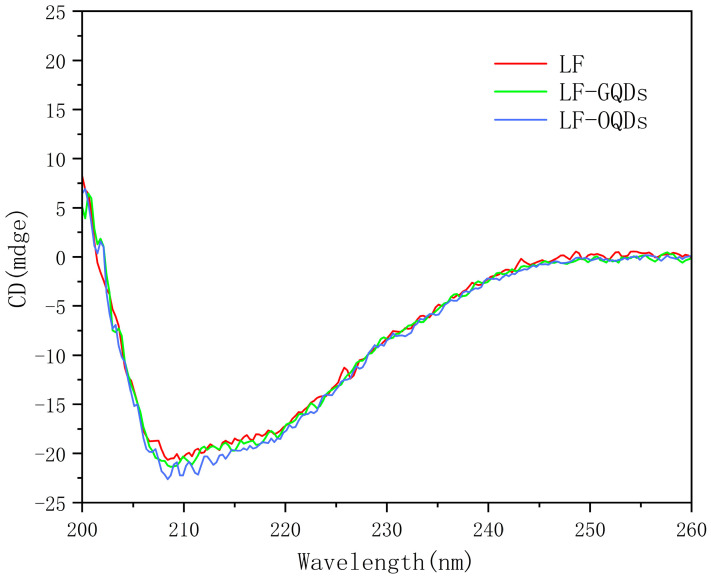
Circular dichroism (CD) spectra of LF and two LF–QDs systems. C(_LF_) = 4.5 × 10^−6^ mol L^−1^; C(_GQDs_) = C(_OQDs_) = 8.0 × 10^−7^ mol L^−1^.

CD has been commonly used as an efficient analytical technique to probe changes in the secondary structure of proteins [28,37]. Generally, the negative peaks at 208 nm and 220 nm are associated with the α-helix of the protein. Figure 12 shows the CD spectra of LF with two LF–QDs systems, and, in order to obtain information about the structure of LF after interaction with QDs, this was calculated using the following equation [38]:

(9) $$MRE\;=\frac{\;Observed\;CD\left(\;mdeg\;\right)}{\left[C_{P}nl\times 10\right]}$$

(10) $$\alpha -helix\left(\%\right)=\left[\frac{-MRE-4000}{33000-4000}\right]\times 100$$

where *MRE* is the ellipticity value measured at 208 nm, *C_p_* is the molar concentration of LF, *n* is the number of amino acid residues in LF, and *l* is the optical path length.

With the addition of both QDs in the LF solution, the secondary structure of LF was changed to different degrees. The α-helix content of LF increased from 31.16% to 32.84% (GQDs) and 35.23% (OQDs), respectively, which indicates that the larger size of the QDs has a greater impact on the biological function of the LF. In addition, the increase of α-helix also indicates the enhanced hydrophobic environment of LF, which is consistent with the results of the above work.

## 3. Materials and Methods

### 3.1. Chemicals

CdCl_2_·2.5H_2_O, Na_2_TeO_3_, NaBH_4_, NaOH, NaCl, and LF were provided by Aladdin Reagent Co., Ltd. (Shanghai, China); GSH was purchased from Saiguo Biotechnology Co. (Shanghai, China); ZnCl_2_ was purchased from Sigma-Aldrich (Shanghai, China); Tris purchased from Biotech Biologicals Co. (Shanghai, China); Anhydrous ethanol was purchased from Xilong Science Co. (Shantou, China); HCl was purchased from Sinopharm Chemical Reagent Co. (Shanghai, China). All chemicals used were of analytical grade, and ultrapure water was used in the experiments. LF as well as QDs were dissolved in Tris-Hcl (0.02 M, pH = 7.20–7.40) for subsequent experiments, respectively.

### 3.2. The Synthesis and Purification of CdTe:Zn^2+^ QDs

The synthesis of CdTe:Zn^2+^ QDs was based on the literature [39] with modifications. Briefly, 0.9 mmol CdCl_2_·2.5H_2_O, 0.1 mmol ZnCl_2_, and 0.3 mmol GSH were loaded into a 250 mL double-necked flask containing 80 mL ultrapure water, and the pH was adjusted to approximately 10.5 with 0.5M NaOH under constant stirring; then 0.2 mmol Na_2_TeO_3_ and NaBH_4_ were placed into this solution. Finally, the solution was reacted in an oil bath at 100 °C with a condensing device attached and by controlling the reflux time (as shown in Figure 1C) to obtain QDs with different fluorescence emission. At the end of the reaction, to remove excess impurities, anhydrous ethanol was added to the reaction mixture to precipitate the QDs. After centrifugation three times, the prepared product was dried overnight under vacuum at 50 °C and stored in a refrigerator at 4 °C for subsequent experiments.

### 3.3. The Characterization of CdTe:Zn^2+^ QDs

The optical properties of the QDs were tested with TU-1901 spectrometer (Beijing Pu-Analysis) and F-4700 fluorescence photometer (Hitachi, Ibaraki, Japan). The morphologyandcrystal structure was investigated with HRTEM (JEM-2010, JEOL, Showashima, Tokyo, Japan) and XRD (Smartlab-3, Rigaku, Akashima, Tokyo, Japan), and the elemental composition of the materials was analyzed using XPS (Thermo Scientific Escalab 250, Thermo Fisher, Franklin, MA, USA). In addition, the surface charge states of QDs and LF were measured using a nanoparticle sizer (Zetasizer Nano ZS 90, Malvern, Worcestershire, UK).

### 3.4. Fluorescence Spectrometry

The fluorescence emission spectra (λ_em_) of two LF–QDs systems were measured at three temperatures (298.15 K, 305.15 K, 313.15 K) on an F-4700 fluorescence spectrometer equipped with a 1.0 cm quartz cassette; the excitation wavelength (λ_ex_) was set to 280 nm, and the excitation width and slit width were both 10 nm. The average of the three scans was taken as the final spectrum. In this process, the LF concentration was 1.0 × 10^−6^ mol L^−1^, and the concentrations of GQDs and OQDs were incremented from 0 to 11.0 × 10^−7^ mol L^−1^.

The 3D fluorescence spectra of LF and two LF–QDs systems were performed under the same spectrometer with the excitation wavelength range set to 200–350 nm and the emission wavelength range set to 200–500 nm in increments of 1 nm. All other scan parameters were the same as those of the steady-state fluorescence spectra. In this process, the LF concentration was 2 × 10^−6^ mol L^−1^, and the concentration of CdTe: Zn^2+^ QDs was 5.0 × 10^−7^ mol L^−1^.

The synchronous fluorescence spectroscopy of the two LF–QDs were measured using the same instrument as above, where Δλ (Δλ = λ_em_ − λ_ex_) was fixed at 15 nm and 60 nm for the measurement of tyrosine residues and tryptophan residues, respectively. The concentrations of LF and QDs were taken to be consistent with the steady-state spectra.

### 3.5. UV–Vis Absorption Spectrometry

For LF as well as for the two LF–QDs systems, UV-Vis absorption spectra were obtained with a TU-1901, a spectrometer equipped with a quartz cuvette with an optical range length of 1 cm, a scan step of 0.5 nm, and a scan range of 200 nm–310 nm. The concentrations of the proteins of both QDs were consistent with those in the steady-state fluorescence spectra.

### 3.6. Circular Dichroism (CD) Spectra Measurements

The CD spectra of LF and two LF–QDs systems were obtained at 298.15 K using a Chirascan circular dichroism instrument (Applied Photophysics Ltd., Surrey, UK). The scanning speed was 200nm/min, the response time was 0.5s, and the wavelength range was 200 to 260 nm. Three consecutive scans were performed for each CD spectrum and averaged.

## 4. Conclusions

In the present work, two particle-sized CdTe:Zn^2+^ QDs were successfully synthesized, and their binding interaction with LF were systematically studied using different spectroscopic methods, including fluorescence spectroscopy, UV-Vis absorption spectroscopy, synchronous fluorescence spectroscopy, 3D fluorescence spectroscopy, and CD spectroscopy for the first time. The results revealed that both sizes of QDs bound strongly with LF with a molar ratio of 1:1 under the main electrostatic force, leading to the static fluorescence quenching of LF. Moreover, the larger size of the QDs brings the interaction distances closer, which reduces the intrinsic fluorescence of LF significantly. In addition, the secondary and tertiary structures of LF are changed to different degrees in the presence of both QDs. This study found that the addition of QDs increases the percent of α-helix of LF (LF: 31.16%, LF–GQDs systems: 32.84%, LF–OQDs systems: 35.23%), which enhanced the hydrophobicity and weakened the biological activity of LF. These results reveal the binding mechanism of the interaction between transition metal-doped, Cd-based QDs and LF at a molecular level, providing useful information for the potential application of Cd-based QD in biological fields.

## Figures and Tables

**Table 1:** The quenching constants (*K_sv_*), quenching rate constants (*K_q_*), and associative binding constants (*K_a_*) of two LF–QDs systems at different temperatures.

System	*T* (K)	*K_sv_* (10^5^ L mol^−1^)	*K_q_* (10^13^ L mol^−1^s^−1^)	R^2^	*K_a_* (10^5^ L mol^−1^)	R^2^
LF–GQDs	298.15	8.24	8.24	0.998	2.74	0.999
	305.15	6.74	6.74	0.996	2.31	0.999
	313.15	4.41	4.41	0.992	1.73	0.999
LF–OQDs	298.15	20.9	20.9	0.997	15.5	0.999
	305.15	17.6	17.6	0.994	12.5	0.999
	313.15	15.6	15.6	0.996	10.5	0.998

**Table 2:** Binding constants (*K*_b_) and binding number (*n*) of two LF–QDs systems.

System	*K_b_* (10^6^ L mol^−1^)	*n*	R^2^	S.D.
LF–GQDs	1.68	1.05	0.998	0.018
LF–OQDs	6.94	1.08	0.996	0.029

R^2^ is the correlation coefficient; SD is the standard deviation.

**Table 3:** Thermodynamic parameters of two LF–QDs systems at different temperatures.

System	*T* (K)	Δ*H* (KJ)	Δ*G* (KJ mol^−1^)	Δ*S* (J mol^−1^ k^−1^)	R^2^
LF–GQDs	298.15	−24.0	−30.8	23.1	0.990
	305.15		−31.0		
	313.15		−31.2		
LF–OQDs	298.15	−16.7	−35.2	62.0	0.999
	305.15		−35.6		
	313.15		−36.1		

**Table 4:** Stern–Volmer quenching constants (*K_SV_*) and associative binding constants (*K_a_*) of two LF–QDs systems in the presence and absence of 0.2 M NaCl at 298.15 K.

System	*K_sv_* (10^5^ L mol^−1^)	R^2^	*K_a_* (10^5^ L mol^−1^)	R^2^
LF–GQDs	8.24	0.998	2.74	0.999
LF–GQDs–NaCl	4.86	0.995	2.36	0.999
LF–OQDs	20.9	0.997	15.5	0.999
LF–OQDs–Nacl	13.8	0.998	9.96	0.999

**Table 5:** Three-dimensional fluorescence spectra of LF and two LF–QDS systems.

System	Peak 1 (λ_ex_/λ_em_)	Intensity	Δλ	Peak 2 (λ_ex_/λ_em_)	Intensity	Δλ
LF	275/331	216.2	56	230/329	63.3	99
LF–GQDs	275/329	155.3	54	230/325	37.1	95
LF–OQDs	275/327	74.5	52	230/319	12.1	89

## Data Availability

All data and materials related to the study are available upon request.
